# Increased conductance of individual self-assembled GeSi quantum dots by inter-dot coupling studied by conductive atomic force microscopy

**DOI:** 10.1186/1556-276X-7-278

**Published:** 2012-05-31

**Authors:** Yifei Zhang, Fengfeng Ye, Jianhui Lin, Zuimin Jiang, Xinju Yang

**Affiliations:** 1 State Key Laboratory of Surface Physics, Fudan University, Shanghai 200433, China

**Keywords:** Conductance, Conductive atomic force microscopy, GeSi quantum dots, Coupling

## Abstract

The conductive properties of individual self-assembled GeSi quantum dots (QDs) are investigated by conductive atomic force microscopy on single-layer (SL) and bi-layer (BL) GeSi QDs with different dot densities at room temperature. By comparing their average currents, it is found that the BL and high-density QDs are more conductive than the SL and low-density QDs with similar sizes, respectively, indicating the existence of both vertical and lateral couplings between GeSi QDs at room temperature. On the other hand, the average current of the BL QDs increases much faster with the bias voltage than that of the SL QDs does. Our results suggest that the QDs’ conductive properties can be greatly regulated by the coupling effects and bias voltages, which are valuable for potential applications.

## Background

Self-assembled semiconductor quantum dots (QDs) have been intensively studied over past decades due to their great importance for both fundamental physics and device applications [1-3]. As the efficiency of single-layer QDs is relatively low, vertically aligned multilayer QDs are often adopted for practical applications [3-6]. By repeating dot layers separated by spacer layers with a few nanometers in thickness, a more homogeneous size distribution could be achieved, simultaneously with novel physical properties induced by coupling [7,8]. The coupling effects between the vertically aligned QDs have been investigated by various macroscopic techniques such as photoluminescence (PL) and admittance spectroscopies [6,8-13], which are found to be strongly dependent on the thickness of the spacer layer. On the other hand, both high-density QDs and QD molecules have attracted a lot of interests for their potential applications [3,14], where the lateral couplings between adjacent QDs significantly modify the QDs’ properties. The lateral coupling effects have also been studied, mainly by macroscopic techniques such as PL spectroscopies [15,16]. Due to the large scattering in QDs’ size, separation, or composition distribution, the quantum properties of coupled QDs obtained by the macroscopic methods would be greatly weakened or eliminated by the averaging effects. Up to now there are only a few microscopic studies performed by STM on InAs [17] and PbSe [18,19] QD clusters recently. In these studies, current–voltage characteristics were found to vary with the dot number in the cluster, indicating the existence of lateral coupling.

Thus the coupling effects between individual QDs from a microscopic viewpoint have been scarcely investigated, let alone the modification of the electrical properties induced by the coupling effects. In this letter, we will employ conductive atomic force microscopy (CAFM) to study the conductive properties of individual GeSi QDs influenced by the QDs’ vertical and lateral couplings at room temperature. CAFM has already been applied to study the conductive properties of individual quantum structures [20-24], but it has rarely been applied to study the coupling effects between individual quantum structures. Here the conductive properties of individual single-layer (SL) and bi-layer (BL) GeSi QDs with different dot densities are investigated by CAFM. Both the vertical coupling between BL QDs and the lateral coupling between densely-packed QDs are found to exist at room temperature, which significantly increase in the QDs’ conductance.

## Methods

The GeSi QDs used for CAFM measurements were fabricated by molecular beam epitaxy on the p-type Si (100) substrate (1 ~ 10 Ω cm). The Si wafers were chemically cleaned by the Shiraki method, and the thin protective oxide was desorbed at 1000°C in ultrahigh vacuum. SL samples A and C were prepared by depositing a 2.9 and 3.4 nm Ge on a 100 nm thick Si buffer layer at the temperature of 640°C, respectively. BL samples B and D were fabricated by depositing another 2.9 and 3.4 nm Ge on samples of A and C respectively, with a 5 nm Si acting as the spacer layer. In previous studies [13,25], the GeSi QDs separated by a 5 nm Si spacer layer were found to exhibit vertical coupling effects. The topography and current measurements were carried out with a commercial AFM equipment (MultiMode V, Bruker Nano Surfaces Division, Santa Barbara, CA, USA) at room temperature. The topographic images of GeSi QDs were obtained by AFM in tapping mode, while their conductive properties were measured by CAFM in contact mode. In CAFM measurements Pt-coated Si tips were used, and the bias voltage was applied to the substrate while the tip was grounded. Before each measurement, the samples were dipped in diluted HF solution for 30 s to remove the oxide layer and to obtain a hydrogen-terminated surface. To sufficiently reduce the influence of local anode oxidation, the current images were measured at negative sample biases and all experiments were performed in a flowing nitrogen atmosphere.

## Results and discussion

**Figure 1:**
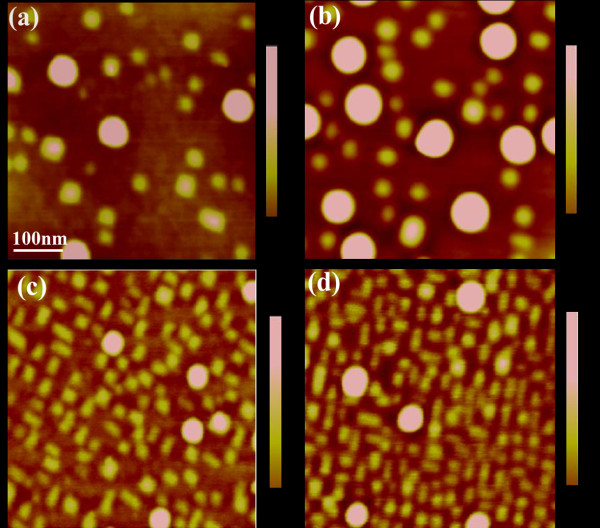
**Tapping mode AFM images of four samples.** (**a**) Single layer/low dot-density sample A, (**b**) bilayer/low dot-density sample B, (**c**) single layer/high dot-density sample C, and (**d**) bilayer/high dot- density sample D. All samples have two types of QDs, and the average sizes of both large and small QDs of the low dot-density samples (A and B) are larger than those of the high dot-density samples (C and D), respectively.

The topographic images of the four samples are shown in Figure1. It can be found that the topographic images of samples A and B (Figures 1 (a) and (b) respectively) are similar, which have two types of QDs. Both large QDs of 50 ~ 70 nm in diameter and small QDs of 30 ~ 50 nm in diameter are observed on samples A/B, except that the density of the large QDs on sample B is higher than that on sample A. On the other hand, the topographic images of samples C and D (Figures 1 (c) and (d) respectively) are also similar, which have two similar types of QDs: large QDs with the diameters of 40 ~ 50 nm and small QDs with the diameters of 20 ~ 30 nm. As the sizes and densities of the QDs are fluctuated, the average sizes of each type of QDs are calculated by doing statistics over a large number of the corresponding QDs, while their densities are obtained by averaging the dot numbers counted from different images. The statistical results of diameters, heights and densities of both large and small QDs of the four samples are listed in Figure1. It can be obtained that samples C/D have higher dot-density but smaller dot-size than samples A/B.

**Table 1:** The statistical results of dot diameters, heights and densities for four samples

**Samples**	**Layers**	**Dot diameter (nm)**		**Dot height (nm)**		**Dot density(/cm**^**2**^**)**	
		**Large dot**	**Small dot**	**Large dot**	**Small dot**	**Large dot**	**Small dot**
Sample A	1	64 ± 5	40 ± 7	6.6 ± 0.8	1.5 ± 0.4	(1.2 ± 0.3) × 10^9^	(1.2 ± 0.5) × 10^10^
Sample B	2	73 ± 6	38 ± 8	8.8 ± 0.6	2.4 ± 0.6	(3.9 ± 0.5) × 10^9^	(1.2 ± 0.5) × 10^10^
Sample C	1	44 ± 6	27 ± 7	5.2 ± 0.7	1.5 ± 0.5	(2.0 ± 0.4) × 10^9^	(6.4 ± 0.9) × 10^10^
Sample D	2	47 ± 6	26 ± 8	5.0 ± 0.9	1.4 ± 0.5	(2.0 ± 0.4) × 10^9^	(7.8 ± 1.0) × 10^10^

**Figure 2:**
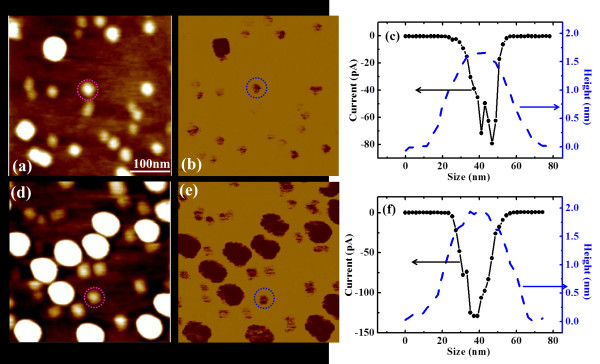
**Topography and current images of sample A/B with height and current profiles of small QDs.** Topography and current images of SL sample A (**a, b**) and BL sample B (**d, e**) with low dot densities. The height and current profiles of the marked small QDs of sample A and sample B are plotted in (**c**) and (**f**), respectively. It shows that the two marked QDs have similar sizes, but the current of the BL QD is about two times larger than that of the SL QD.

The conductive properties of all samples are measured by CAFM at different sample biases. The current images measured on samples A (SL) and B (BL) at the bias voltage of −0.5 V are shown in Figure2, together with their corresponding topographic images. For both samples, large current is measured on the QDs compared with the wetting layer, indicating that the QDs are more conductive than the wetting layer. In addition, the current measured on large QDs are larger than that measured on small QDs, which means that, the current increases with the dot size. Similar phenomenon was observed by Tanaka et al. on InAs QDs [26], where they found the conductance on InAs QDs were larger than that on the wetting layer and attributed it to the band lowering effect by surface states on the InAs QDs. As our results of GeSi QDs are very similar to that of InAs QDs, we applied the above concept to explain why the GeSi QDs are more conductive than the wetting layer, and why the larger QDs are more conductive by the assumption of the larger band lowering effects on larger QDs. By comparing the current images of samples A and B (Figures 2 (b) and (d)respectively), it can be found that the average current of the BL QDs is much larger than that of the SL QDs with similar sizes. The typical current profiles of single small QDs for SL and BL samples (marked by circles) are plotted in Figures 2 (c) and (f), respectively, together with their corresponding height profiles. It can be seen that the sizes of the two QDs are similar (~ 50 nm in diameter and ~1.8 nm in height), but the current values of the BL QD are about two times that of the SL QD. For large QDs (profiles are not shown here), it can be found that the current values of the BL QDs are also larger than their SL counterparts. Thus the BL QDs are much more conductive than the SL QDs, for both large and small QDs. 

**Figure 3:**
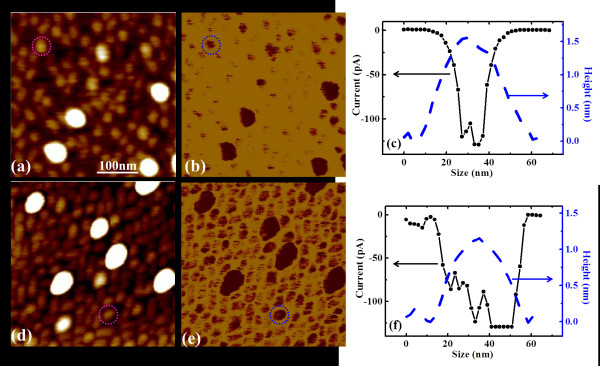
**Topography and current images of samples C/D with height and current profiles of small QDs.** Topography and current images of SL sample C (**a,b**) and BL sample D (**d,e**) with high dot densities. The height and current profiles of the marked small QDs of sample C and sample D are plotted in (**c**) and (**f**), respectively. It shows that the BL QD has smaller size but larger current than the SL QD.

The same conclusion could be drawn by comparing the conductive results of high-density samples C (SL) with D (BL), which are shown in Figure3. From the current images of samples C and D (Figures 3(b) and 3(e) respectively), it could be found that the BL QDs are also more conductive than the SL QDs, for both large and small QDs. A better comparison can be achieved in the height and current profiles of single typical small QDs for SL and BL samples as shown in Figures 3(c) and 3(f) respectively. The current values of the BL QD are larger than those of the SL QD with the similar size, where the current on a large part of the BL QD is saturated due to the measurement limitation.

To sum up, our results indicate that the BL QDs are more conductive than SL QDs with similar size for both low and high dot densities, but the origin is not clear yet. In CAFM measurements, as the area of the current flow increases fast along the current path, the major contribution to the current is the certain surface region which contacts with the tip. Hence without the vertical coupling, the second-layer QDs will not influence the current. Therefore a possible mechanism is assumed in terms of the tunneling effects between the coupled QDs. Due to the vertical coupling, the density of states which contribute to the electron tunneling would be larger than those of the single QD. Thus the QDs in the second layer can contribute to the conductance through the vertical coupling, which makes the QDs in the first layer more conductive.

On the other hand, the influence of QDs’ density on their conductive properties is also concerned. By comparing the current images of different-density SL samples (Figures 2(b) and 3(b)) and BL samples (Figures 2(e) and 3(e)), it is found that the higher the dot density, the larger the average current of the QDs with similar sizes. It should be mentioned that the influence of current by the QD’s density is not as significant as that by the layer effect. The possible reason may be due to the differences of the QDs’ size, where the sizes of both the large and small QDs of higher-density samples C/D are smaller than those of the lower-density samples A/B, respectively. As the current of QDs decreases with the dot size decreasing, the increase of the current with dot density will be hindered by the decrease of dot size. Nevertheless, it can still be found that the average current of QDs with high dot-density is larger than that of the QDs with low dot-density, for both large and small QDs and for both SL and BL QDs. As the current measured by CAFM only comes from the contact area between the tip and its beneath surface and the contact area is smaller than the area of a single QD, the nearby dots will not influence the measured current without the lateral coupling. Thus our results indicate the existence of the lateral coupling between the closely packed QDs at room temperature, which increases the QDs’ conductance. It should be mentioned, though the dot-density of the large QDs on sample C/D is not large enough for coupling, the large QDs can still couple with the small nearby QDs, which can also increase their conductance. Similar lateral coupling effects have been observed on InAs or PbSe QD clusters by STM [16-19]. The measured current was found to increase with the dot number, which was interpreted by the increasing of tunneling path when QDs were closely packed. Our results are consistent with the STM observations, thus the above interpretation can be adopted to explain our results. For high-density QD samples, the conductive path between the tip and the sample increases, i.e. electron tunneling between lateral coupled QDs, resulting in the increased conductance.

**Figure 4:**
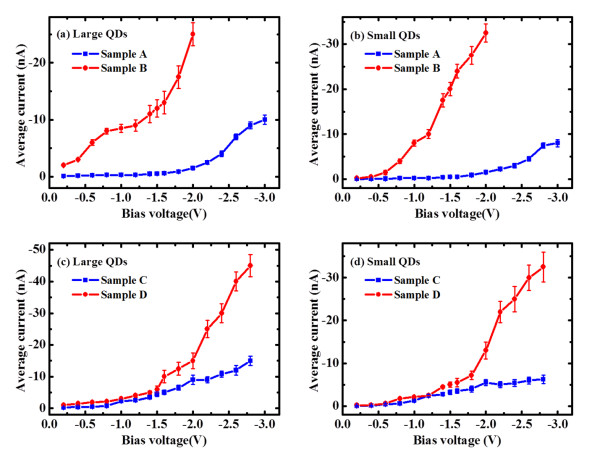
**The obtained average current as a function of bias voltage.** (**a**) Large QDs of samples A/B, (**b**) small QDs of samples A/B, (**c**) large QDs of samples C/D, and (**d**) small QDs of samples C/D.

In addition, the influences of inter-dot coupling on the QDs’ conductance are investigated as a function of bias voltage. The bias dependencies of the average current, which is deduced from the peak current in the histogram of current magnitude by calculating over a number of QDs with similar sizes, are shown in Figure4. By comparing the results of the large and small QDs of sample A with those of sample B, it can be found that the average current values of BL QDs are larger than those of their corresponding SL QDs at all biases, for both large and small QDs (Figures 4(a) and 4(b) respectively). Additionally the increased ratio of BL QDs to SL QDs increases with the bias voltage, resulting in significant increases of the conductance for BL QDs at large biases. For example, the average current of large and small QDs of the BL sample is about 17 and 21 times of their corresponding SL ones at −2 V, respectively. Similar increases of the conductance for BL QDs over SL QDs are observed on the high-density samples C/D, for both large and small QDs (Figures 4(c) and 4(d) respectively). For example, the increased ratio of BL QDs to SL QDs at −2.6 V is about 3 and 5 times for large and small QDs, respectively.

On the other hand, the increase of the conductance by the dot density can be observed on SL QDs of samples A/C. By comparing the averaging currents of large QDs (sample A in Figure4(a) and sample C in Figure4(c)), as well as those of small QDs (sample A in Figure4(b) and sample C in Figure4(d)), the current increased ratios of high-density QDs (sample C) to low-density QDs (sample A) also increased with the bias voltage, which are about 6 and 4 times at −2 V for large and small QDs respectively. By considering the size effect, the increased ratio should be even larger. For BL samples B/D, however, the increase of conductance by dot density could not be observed, for both large QDs (Figures 4(a), (c)) and small QDs (Figures 4(b), (d)). The reason is not clear yet, which may be due to the already existed vertical coupling between BL QDs. The large increase of QDs’ conductance at large biases should be an exciting result, as it suggests that the coupled QDs’ conductive properties can be greatly regulated by bias voltage, which should be valuable for applications. The bias dependence of the conductance of individual QDs has been investigated in our previous paper [27]. It was found that the QDs’ current increases much faster with the bias than the wetting layer, which was attributed to the discrete energy levels of QDs. With the similar concept, the larger bias-dependence of the conductance of the coupled QDs may be also attributed to the energy levels of the coupled QDs.

## Conclusions

In summary, the influences of both the vertical and lateral couplings on the conductive properties of individual GeSi QDs are studied by CAFM at room temperature. By comparing the current images of SL and BL QDs with different dot densities, it could be found that for the QDs with similar sizes, the BL QDs are much more conductive than the SL QDs for both low and high dot densities, and the high-density QDs are more conductive than the low-density QDs for both SL and BL samples. In addition, the average current of the BL QDs increases much faster with the bias voltage than the SL QDs, resulting in large conductance increases of BL QDs over SL QDs at large biases. From the above results, we suggest that both the vertical and lateral couplings between individual GeSi QDs exist at room temperature, which significantly enhance the QDs’ conductance.

## Competing interest

The authors declare that they have no competing interests.

## Authors’ contributions

YFZ carried out the experiments. FFY participated in the CAFM measurements. JHL prepared the samples. YFZ and XJY interpreted the results and wrote the manuscript. ZMJ participated in sample preparation and helped in manuscript preparation. All authors read and approved the final manuscript.
